# Possibility of γδ T cell immunotherapy for HTLV-1-infected individuals

**DOI:** 10.1186/1742-4690-8-S1-A109

**Published:** 2011-06-06

**Authors:** Tomoo Sato, Masato Muto, Natsumi Araya, Ryuji Maekawa, Noboru Suzuki, Atae Utsunomiya, Ken-ichiro Seino, Yoshihisa Yamano

**Affiliations:** 1 Institute of Medical Science, St. Marianna University School of Medicine, Kawasaki, Japan; 2 Medinet Medical Institute, MEDINET Co. Ltd., Tokyo, Japan; 3 Department of Hematology, Imamura Bun-in Hospital, Kagoshima, Japan; 4 Institute for Genetic Medicine Research Section of Pathophysiology, Hokkaido University, Hokkaido, Japan

## 

γδ T cells, a small subset of T lymphocytes, are involved in innate immunity. It has been demonstrated that γδ T cells have cytotoxic activities against cells infected with a variety of viruses. However, there is little evidence suggesting a cytotoxic activity of γδ T cells against HTLV-1-infected cells. Therefore, we investigated whether γδ T cells play a protective role in the defense against HTLV-1.

Using PBMCs from asymptomatic carriers (ACs) and HAM/TSP patients, we assayed the frequency of CD3+Vγ9+ (γδ T) cells and the correlation between its frequency and the HTLV-1 load. The frequency of γδ T cells was significantly decreased in HAM/TSP patients compared with that in ACs. The frequency of γδ T cells was inversely correlated with the proviral load. These results suggest that γδ T cells have a protective effect on HTLV-1-infected individuals. Next, CD3+Vγ9+ cells and CD3+Vγ9- cells were separated from PBMCs of HTLV-1-infected persons by FACS and the proviral load of each population was quantified by real-time PCR. The proviral load in γδ T cells was markedly lower than that in CD3+ lacking γδ T cells. Furthermore, we cultured PBMCs from HTLV-1-infected individuals in the presence of IL-2 and zoledronate. In some cases, the majority of cells contained in these cultures became γδ T cells and the proviral load was markedly decreased. The cultured PBMCs showed strong cytotoxic activities against a HTLV-1-infected cell line as well as an ATL cell line. These results raise the possibility of γδ T cell immunotherapy in HTLV-1-infected individuals.

